# Crosstalk in the kidney-muscle axis: myokines and muscle-relevant mediators in chronic kidney disease-associated sarcopenia

**DOI:** 10.3389/fmed.2026.1868285

**Published:** 2026-06-12

**Authors:** Ran Hu, Xuelian Zhang, Lan Hu, Xu Li, Qin Hu, Hua Jin

**Affiliations:** Department of Nephrology, The First Affiliated Hospital of Anhui University of Chinese Medicine, Hefei, China

**Keywords:** chronic kidney disease, kidney-muscle axis, muscle wasting, myokines, protein-energy wasting, renal clearance, sarcopenia

## Abstract

Chronic kidney disease (CKD) is a systemic disorder in which sarcopenia serves as a critical driver of frailty and mortality. However, the “kidney-muscle axis” remains conceptually fragmented, often confounded by the overlapping definitions of protein-energy wasting (PEW) and cachexia. This review argues that CKD-associated sarcopenia is not driven by isolated myokines, but rather by a clearance-distorted, inflammation-coupled signaling network. We first disambiguate sarcopenia from PEW and cachexia, distinguishing canonical myokines from mediators whose interpretive value is altered by uremia. We then propose a framework organized around four pillars: hypercatabolism, anabolic resistance, mitochondrial dysfunction and bioenergetic remodeling, and context-dependent inflammatory signaling. Within this context, we reinterpret key mediators, including myostatin, growth differentiation factor 15 (GDF-15), insulin-like growth factor 1 (IGF-1), irisin, and interleukin-6 (IL-6), emphasizing that their circulating levels reflect a complex entanglement of altered secretion, impaired renal clearance, and tissue-specific resistance. While the kidney-to-muscle vector is well-supported, direct muscle-to-kidney feedback remains less established. By framing myokine dysregulation as a mechanistic interface, this review aims to refine causal inference and support the development of targeted therapies for muscle wasting in CKD.

## 1 Introduction

Chronic kidney disease (CKD) is a major global health burden, affecting approximately 10–15% of adults worldwide. The global burden of CKD continues to increase with population aging and the growing prevalence of diabetes, hypertension, and cardiometabolic diseases (1–3). Beyond progressive renal decline, CKD precipitates diverse systemic complications, among which sarcopenia prominently features as a prevalent, clinically debilitating condition, particularly in end-stage kidney disease (ESKD). It is closely associated with frailty, impaired physical function, hospitalization, reduced quality of life, and increased mortality (4–8). These findings highlight muscle dysfunction as a central component of CKD pathology and a key predictor of patient outcomes (7, 8).

Previous studies have identified inflammation, uremic toxin accumulation, metabolic acidosis, oxidative stress, endocrine resistance, impaired regeneration, and physical inactivity as contributors to CKD-associated sarcopenia (9–17). However, the growing interest in inter-organ signaling has also introduced conceptual complexity into this field. Canonical myokines, non-classical muscle-derived factors, and broader systemic stress mediators are often discussed together, despite important differences in tissue origin, biological context, and dependence on renal clearance (17–19). Similarly, sarcopenia is frequently used interchangeably with protein-energy wasting (PEW) or cachexia, despite their distinct clinical definitions and only partial mechanistic overlap (4, 7, 8, 10).

This review frames CKD-associated sarcopenia as a systems-level remodeling of the kidney-muscle signaling interface rather than as the effect of isolated mediators. The function of this interface is shaped by metabolic stress, inflammation, endocrine resistance, and impaired renal clearance. Within the uremic milieu, individual mediators may serve as direct catabolic signals, context-dependent adaptive responses, biomarkers of systemic burden, or combinations of these roles. Consequently, circulating concentrations may not reliably reflect intramuscular bioactivity, as secretion, systemic accumulation, and renal clearance can diverge substantially in chronic renal failure (9–19).

On this basis, this review organizes CKD-associated sarcopenia around four interrelated pathological processes: hypercatabolism, anabolic resistance, metabolic and mitochondrial dysfunction, and context-dependent inflammatory signaling. This process-centered framework interprets mediators according to their functional roles rather than as a simple molecular catalog. It distinguishes factors more directly linked to catabolic signaling, such as myostatin and activin A, from stress-responsive mediators such as FGF21, which may mainly reflect systemic wasting burden (13–19). Similarly, mediators such as IL-15 and IL-6 require context-specific interpretation, as their biological effects depend on tissue responsiveness, source, timing, and the broader uremic milieu.

This review also refines the conceptual scope of the kidney-muscle axis. Although reciprocal crosstalk is biologically plausible, CKD-specific evidence currently supports kidney-to-muscle signaling more strongly than direct muscle-to-kidney signaling (17, 18). We therefore use the “kidney-muscle axis” to describe how renal dysfunction reshapes the systemic environment that regulates muscle homeostasis. We aim to clarify the distinctions among sarcopenia, PEW, and cachexia; distinguish canonical myokines from broader circulating mediators; organize current evidence around hypercatabolism, anabolic resistance, metabolic and mitochondrial dysfunction, and pleiotropic signaling; and separate biomarker associations from causal mechanisms by considering tissue origin, evidence strength, and impaired renal clearance.

## 2 Conceptual scope of the kidney-muscle axis in CKD-associated sarcopenia

### 2.1 Distinguishing sarcopenia, PEW, and cachexia

Conceptual ambiguity in the CKD literature partly arises because sarcopenia, PEW, and cachexia overlap but are not equivalent. Sarcopenia is fundamentally defined by the loss of muscle mass paired with diminished muscle strength or impaired physical performance (4, 6–8). Contemporary consensus frameworks now anchor sarcopenia more firmly to functional deficits such as low strength and reduced performance rather than to lean mass alone (20, 21). In contrast, PEW is a broader, CKD-specific syndrome that encapsulates the depletion of body protein and energy stores. This condition is driven by a multifactorial etiology including reduced dietary intake, systemic inflammation, endocrine disturbances, and catabolic activation (10, 22). Cachexia shares features with both conditions but typically denotes a more advanced systemic wasting state characterized by involuntary weight loss, anorexia, and high-grade inflammatory activation (23). While these syndromes frequently coexist in the CKD population, they represent distinct clinical entities and should not be used interchangeably. This distinction is critical when interpreting mediators such as GDF-15 and FGF21, which may be more closely related to anorexia, energy imbalance, and systemic wasting than to isolated sarcopenia (10, 24–38).

### 2.2 Conceptualizing myokines and muscle-relevant mediators

Strictly defined, myokines are cytokines, peptides, or hormone-like factors synthesized and secreted by skeletal muscle that exert their effects through autocrine, paracrine, or endocrine pathways (17–19). In CKD, however, sarcopenia is modulated by a much broader signaling milieu that includes factors with diverse or context-dependent tissue origins. To ensure conceptual clarity, this review adopts a two-tier framework. Canonical myokines discussed herein primarily include myostatin, IL-15, irisin, and exercise-induced IL-6. In contrast, GDF-15, FGF21, and brain-derived neurotrophic factor (BDNF) are categorized as muscle-relevant circulating mediators. These factors may influence muscle metabolism, mitochondrial adaptation, appetite regulation, and physical function in CKD, but their primary source of production is not exclusively skeletal muscle (24–46). This nuanced classification avoids an overly rigid taxonomy while maintaining the biological precision necessary for mechanistic discussion.

We use the term “kidney-muscle axis” in a focused mechanistic sense. Evidence suggests that renal dysfunction reshapes the systemic signaling environment primarily through chronic inflammation, uremic toxin retention, endocrine resistance, metabolic acidosis, and the impaired clearance of circulating mediators (9–16, 47, 48). Although reciprocal signaling is biologically plausible, CKD-specific evidence that muscle-derived signals directly protect or damage the kidney remains limited (17, 18). Therefore, this review retains a bidirectional framework but emphasizes that the kidney-to-muscle direction is currently better supported by evidence.

## 3 Core biological processes linking CKD to muscle loss

Before discussing individual mediators, we first outline the major biological processes underlying CKD-associated sarcopenia. First, CKD disrupts the fundamental proteostatic balance between synthesis and degradation. Under physiological conditions, anabolic signaling orchestrated via the IGF-1/PI3K/Akt/mTORC1 axis drives ribosomal biogenesis and *de novo* muscle protein synthesis (49, 50). In the uremic milieu, this pathway is blunted by the accumulation of IGF-binding proteins, post-receptor defects involving IRS-1, pro-inflammatory signaling, and systemic insulin resistance, all of which collectively manifest as a state of anabolic resistance (14–16). Concurrently, protein degradation increases through FoxO-dependent transcription, activation of the ubiquitin-proteasome system (UPS), and autophagy-mediated proteolysis (13, 51–54).

Second, CKD compromises skeletal muscle regenerative capacity. While satellite cells remain the primary engine for myofiber renewal and adaptive remodeling, their activation, proliferation, and myogenic differentiation are impaired by chronic inflammation, oxidative stress, uremic toxin exposure, and dysregulated myokine signaling (13, 55–57). Thus, CKD-associated muscle wasting reflects not only accelerated proteolysis but also impaired compensatory repair.

**Figure 1:**
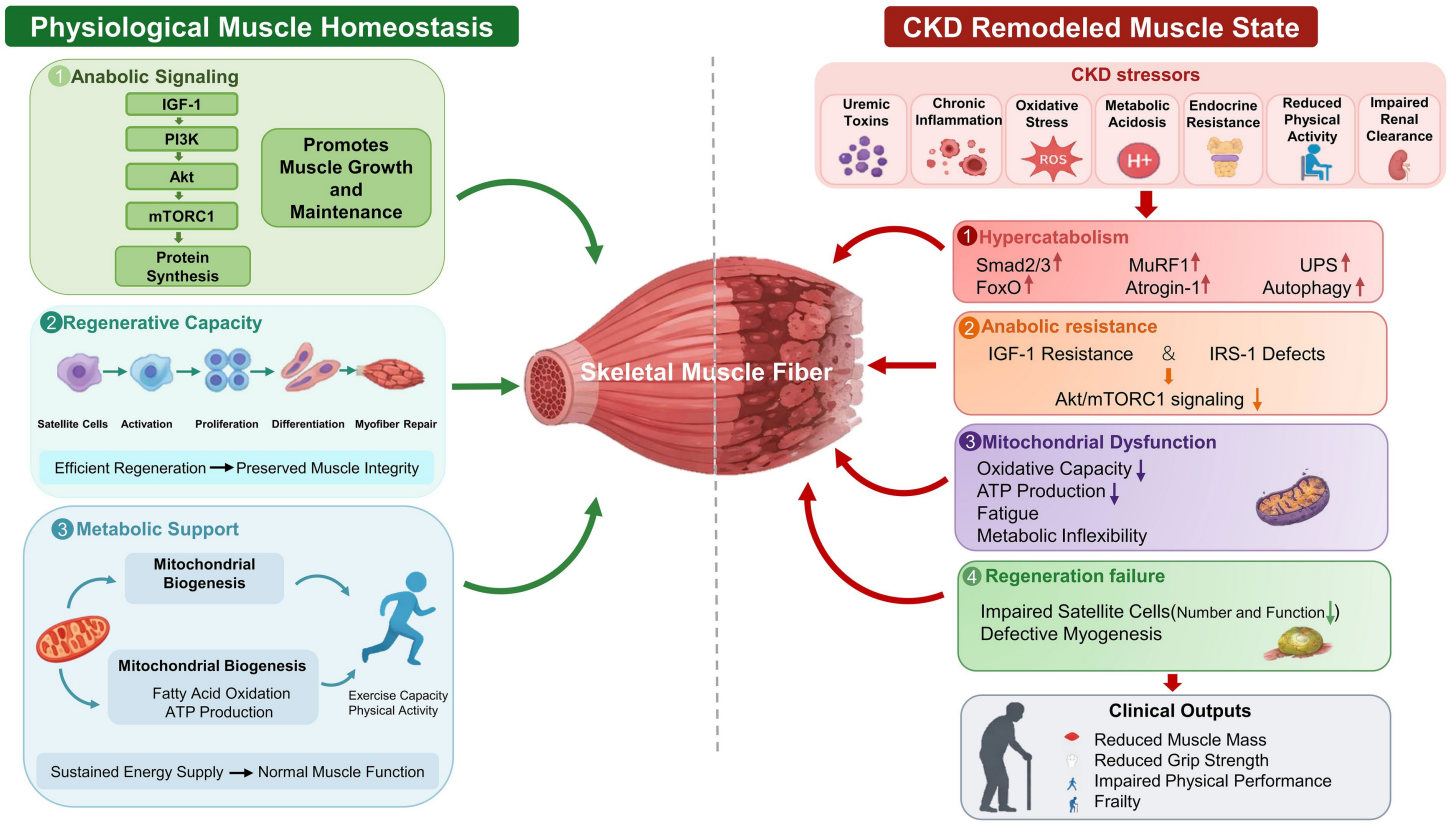
Key anabolic, regenerative, and inhibitory processes governing skeletal muscle maintenance in CKD-associated sarcopenia. This figure summarizes the major biological processes that maintain skeletal muscle homeostasis under physiological conditions and how they are disrupted in CKD. Under normal conditions, anabolic signaling through the IGF-1/PI3K/Akt/mTORC1 axis supports protein synthesis and muscle maintenance, while satellite cell activation and differentiation preserve regenerative capacity. Mitochondrial biogenesis and oxidative metabolism further sustain contractile function and exercise tolerance. In CKD, this balance is progressively disrupted by uremic toxin retention, chronic inflammation, oxidative stress, metabolic acidosis, endocrine resistance, and physical inactivity. These CKD-related stressors suppress anabolic signaling, activate FoxO-dependent and ubiquitin-proteasome/autophagy-mediated proteolysis, impair mitochondrial function, and blunt satellite-cell-dependent regeneration. The combined result is a shift toward hypercatabolism, anabolic resistance, mitochondrial dysfunction, and regeneration failure, ultimately leading to reduced muscle mass, strength, and physical performance.

Third, mitochondrial dysfunction and bioenergetic remodeling are central to the wasting phenotype. Factors including uremic toxins, physical inactivity, and disordered endocrine signaling synergistically impair mitochondrial biogenesis, fatty acid oxidation, and metabolic flexibility (11, 12, 14, 15). The resulting attrition in oxidative capacity diminishes exercise tolerance and exacerbates physical inactivity, thereby establishing a self-perpetuating cycle that accelerates skeletal muscle wasting. By framing the discussion around these core pillars, the diverse roles of myokines and circulating mediators can be integrated into a coherent pathophysiological model. These core biological processes are summarized in Figure 1.

## 4 Myokines and muscle-relevant circulating mediators in CKD-associated sarcopenia

**Figure 2:**
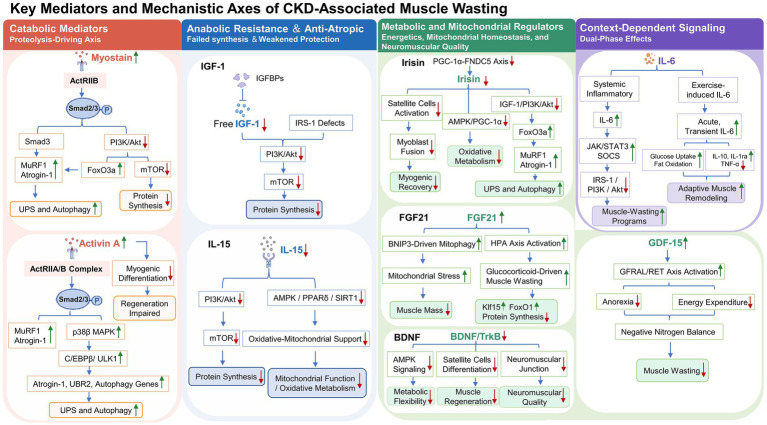
Key mediators and mechanistic axes of CKD-associated sarcopenia. Catabolic and anabolic-resistance pathways: myostatin and activin A promote proteolysis and impair muscle regeneration via ActRIIB/ActRIIA-Smad2/3 and p38β MAPK signaling. Conversely, impaired IGF-1 and IL-15 signaling weakens anabolic and oxidative-mitochondrial support. Metabolic and mitochondrial regulators: altered irisin, FGF21, and BDNF signaling disrupt homeostatic networks, linking mitochondrial dysfunction, proteostatic imbalance, and defective myogenesis to neuromuscular decline. Additionally, GDF-15 may contribute to systemic wasting through GFRAL/RET-mediated appetite suppression and energy-balance disruption. Context-dependent inflammatory signals: IL-6 exhibits distinct source- and time-dependent effects. Chronic systemic IL-6 drives inflammatory catabolism, whereas acute, exercise-induced IL-6 supports metabolic adaptation and tissue remodeling.

Figure 2 summarizes the major catabolic, anabolic-resistance, metabolic and mitochondrial, and inflammatory axes through which myokines and muscle-relevant circulating mediators may contribute to CKD-associated sarcopenia.

### 4.1 Catabolic mediators

#### 4.1.1 Myostatin

Myostatin serves as a potent negative regulator of muscle growth and is primarily synthesized and secreted by skeletal muscle cells, thereby functioning as a canonical myokine (13, 51, 53, 54). While skeletal muscle remains its predominant source, the systemic environment in CKD significantly modulates its bioavailability. In the uremic milieu, elevated circulating myostatin may reflect both increased production driven by inactivity, inflammation, oxidative stress, and glucocorticoid signaling, and impaired renal clearance (13, 47, 51). Notably, the observation that plasma accumulation does not always correlate with a proportional increase in intramuscular transcription suggests that reduced renal excretion is a critical and distinct contributor to its systemic accumulation in patients with CKD (47).

Mechanistically, myostatin promotes muscle atrophy primarily through canonical activin receptor signaling and its downstream crosstalk with anabolic pathways (51). After binding activin type II receptors, with a preference for ActRIIB, and recruiting ALK4/5, myostatin activates Smad2/3 signaling (58, 59). Activated Smad2/3, particularly Smad3, drives a catabolic transcriptional program that directly induces the E3 ubiquitin ligase atrogin-1, thereby activating the UPS to promote muscle protein degradation (60). In parallel, Smad2/3 signaling engages in inhibitory crosstalk with the IGF-1/PI3K/Akt/mTOR axis (51). While this process attenuates Akt-mediated repression of FoxO transcription factors, thereby promoting the expression of atrogenes such as MuRF1, it simultaneously suppresses mTOR-mediated anabolic signaling, leading to reduced muscle protein synthesis (60).

By reinforcing both the ubiquitin-proteasome and autophagy-lysosome pathways, myostatin shifts muscle metabolism toward a state of net protein loss (52). Preclinical studies in CKD models further support a causal role, as myostatin inhibition has been shown to improve muscle mass, grip strength, and anabolic signaling (61, 62). Nevertheless, most clinical trials of anti-myostatin strategies have been conducted outside CKD populations, so CKD-specific translation remains uncertain (63).

#### 4.1.2 Activin A

Activin A shares fundamental signaling architecture with myostatin but possesses a more diverse regulatory profile involving renal pathology, systemic fibrosis, and chronic inflammation (48, 64–66). Unlike canonical myokines that originate predominantly from muscle, Activin A in the context of CKD emanates from both the local muscle environment and, crucially, injured renal tissue (47, 48, 65). As kidney function deteriorates, this dual-source production coupled with impaired renal excretion leads to significant systemic accumulation, which positions Activin A as a quintessential component of the kidney-to-muscle endocrine axis.

Mechanistically, Activin A utilizes ActRIIA/B receptor complexes to trigger canonical Smad2/3 signaling, thereby initiating a catabolic transcriptional program that drives the expression of E3 ligases including atrogin-1/MAFbx and MuRF1 and accelerates protein degradation (67–69). Parallel to this canonical route, Activin A engages a non-canonical p38β MAPK branch that activates the transcription factor C/EBPβ. This secondary pathway preferentially augments the expression of atrogin-1/MAFbx and UBR2 (68) while simultaneously promoting autophagy-related signaling via LC3/Gabarapl1 induction and ULK1-dependent autophagosome formation (70). Notably, the p38β MAPK contribution appears to exert a more profound influence on autophagy activation and the specific upregulation of atrogin-1/UBR2 than on MuRF1 expression.

Beyond acute catabolism, chronic or pathological Activin A signaling impairs myogenic differentiation and may compromise regenerative capacity, though these outcomes appear sensitive to the specific biological context, tissue source, and disease phase (71, 72). Given that Activin A is deeply embedded in pro-inflammatory and fibrotic biology (73, 74), and considering that broad ActRII blockade can pleiotropically affect hematological and bone metabolism (75), future therapeutic strategies must distinguish between global receptor antagonism and more selective targeting of either Activin A itself or its non-canonical p38β MAPK branch.

### 4.2 Mediators of anabolic resistance and anti-atrophic defense

#### 4.2.1 IGF-1

Insulin-like growth factor 1 (IGF-1) is the canonical anabolic regulator of skeletal muscle homeostasis (49). Under physiological conditions, circulating IGF-1 is produced predominantly by the liver under growth hormone control and acts as a systemic endocrine factor. In addition, skeletal muscle cells produce IGF-1 locally, where it functions as an important autocrine and paracrine regulator of muscle mass (76, 77). In CKD, this local IGF-1 axis appears to be disrupted. In sedentary patients receiving chronic hemodialysis, skeletal muscle mRNA expression of IGF-IEa, IGF-II, and IGF-I receptor is reduced, suggesting impaired local anabolic capacity in human uremic muscle (78). Experimental studies further show that uremia attenuates growth hormone-stimulated skeletal muscle IGF-1 expression, an effect that is aggravated by inflammation (79). Thus, the main abnormality of the IGF-1 system in CKD is reduced anabolic activity caused by altered local production, limited ligand availability, and impaired post-receptor signaling.

After binding to its receptor, IGF-1 promotes tyrosine phosphorylation of IRS-1, recruitment of PI3K, and activation of the Akt/mTORC1 axis, thereby stimulating translation initiation, ribosome biogenesis, and myofiber hypertrophy (14, 49, 50). In CKD, this signaling cascade is weakened through two interrelated mechanisms. First, accumulated IGF-binding proteins reduce the free IGF-1 fraction and limit receptor access (15). Consistent with this mechanism, dialysis patients show an impaired metabolic response to recombinant IGF-1, supporting the presence of systemic IGF-1 resistance in advanced kidney disease (80). Second, skeletal muscle post-receptor signaling is defective. In chronic renal failure models, IGF-1 has a reduced ability to stimulate muscle protein synthesis and suppress protein degradation, providing direct evidence for impaired IGF-1 action beyond ligand availability (81). CKD also impairs IRS-1/PI3K/Akt signaling in skeletal muscle, with reduced IRS-1-associated PI3K activity and Akt activation contributing to accelerated muscle protein catabolism (82). Mechanistically, toxin accumulation, chronic inflammation, metabolic acidosis, and insulin resistance promote abnormal serine phosphorylation of IRS-1 in CKD. This modification interferes with downstream signal propagation, weakens PI3K/Akt activation, suppresses mTORC1-mediated protein synthesis, and facilitates FoxO-dependent catabolic programs (16).

Consequently, mTORC1-driven anabolic output may remain insufficient even when nutritional substrates are available. This helps explain why nutritional supplementation alone often fails to reverse muscle wasting in CKD. The limiting factor is not simply substrate supply, but an anabolic signaling network rendered resistant to stimulation. Within the kidney-muscle axis, IGF-1 should therefore be understood as a central anabolic pathway whose effectiveness is constrained by CKD-associated anabolic resistance.

#### 4.2.2 Il-15

Interleukin-15 (IL-15) is a muscle-associated cytokine with anti-atrophic, metabolic, and mitochondrial effects on skeletal muscle (83–85). Under physiological conditions, IL-15 is primarily expressed within skeletal muscle, where it serves as a quintessential myokine, though it is also synthesized by various immune cells and certain non-muscle tissues including the kidneys (86). Its interpretation in CKD is particularly complex due to discordant observations across different biological compartments. While local expression may be significantly reduced in injured renal tissue or fibrosis-associated environments, circulating IL-15 levels often appear elevated in CKD cohorts. This elevation is likely driven by systemic immune activation coupled with reduced renal clearance, highlighting the potential discrepancy between circulating abundance and local biological effectiveness within skeletal muscle (87, 88).

Functionally, IL-15 supports protein synthesis and mitochondrial oxidative capacity through the recruitment of PI3K/Akt/mTOR signaling and the activation of key transcriptional regulators such as PGC-1α (83–85). Overexpression studies demonstrate that IL-15 increases the expression of PPARδ, SIRT1, and both PGC-1α and PGC-1β, thereby promoting a more oxidative myofiber phenotype. In muscle cells, IL-15 enhances mitochondrial activity through a PPARδ-dependent mechanism and induces the expression of Nrf1 along with other coactivators (84, 89). Furthermore, acute exposure to IL-15 has been linked to the activation of the AMPK pathway, which subsequently improves oxidative metabolism and energy homeostasis (85, 90).

In CKD, the critical inquiry is not merely whether circulating IL-15 is high or low, but rather whether target tissues maintain sensitivity to its beneficial actions. This distinction explains why IL-15 is best discussed within the framework of anabolic resistance instead of being categorized solely by absolute changes in concentration. The mismatch between systemic abundance and local signaling efficacy underscores the systemic impairment of anabolic pathways in renal failure.

### 4.3 Metabolic and mitochondrial regulators

#### 4.3.1 Irisin

Irisin remains a compelling link between muscle endocrine activity and mitochondrial homeostasis, proteostasis, regeneration, and physical function in CKD (91–93). It is generated through proteolytic cleavage of FNDC5 and is classically linked to exercise-responsive PGC-1α signaling (91, 94). In CKD, circulating irisin concentrations are generally reduced, and lower levels are associated with poorer renal function, insulin resistance, increased risk of PEW and frailty, and muscle wasting (92, 93). The uremic toxin indoxyl sulfate has been implicated in suppression of the PGC-1α/FNDC5 axis, providing a plausible kidney-to-muscle mechanism underlying irisin deficiency (11, 92). Consistent with this broader uremic toxin milieu, indoxyl sulfate burden has also been associated with reduced handgrip strength and increased hospitalization risk in end-stage renal disease, and may further blunt the protective effects of irisin in CKD (95).

Biologically, irisin appears to act through several mechanisms directly relevant to CKD-associated sarcopenia. In skeletal muscle, irisin supports mitochondrial biogenesis and oxidative metabolism through an AMPK/PGC-1α-centered program and may limit mitochondrial fission and oxidative stress through AMPK/Drp1-related signaling (94, 96–99). It also suppresses proteolysis by enhancing IGF-1/PI3K/Akt signaling and restricting FoxO-dependent induction of the E3 ubiquitin ligases atrogin-1 and MuRF1 (97, 98). In addition, emerging evidence suggests that irisin can promote satellite cell activation, myoblast fusion, and myogenic recovery, indicating that its role extends beyond metabolic support to regenerative competence (99, 100). Given its close relationship with exercise, irisin provides a useful conceptual bridge between molecular pathophysiology and rehabilitation-based intervention. Although its classical browning and thermogenic effects remain important components of its broader biology, its most directly relevant roles in CKD may involve mitochondrial maintenance, suppression of proteolysis, myogenesis, and preservation of physical function.

#### 4.3.2 FGF21

FGF21 is better regarded as a circulating metabolic mediator relevant to skeletal muscle rather than a canonical myokine, because it is produced predominantly by extra-muscular tissues, particularly the liver (32, 40). In CKD, circulating FGF21 levels increase as renal function declines, likely reflecting impaired renal clearance and increased production driven by inflammation and metabolic stress (33, 34). Higher FGF21 concentrations have been associated with poorer muscle status and increased mortality in hemodialysis cohorts (38).

However, the biological interpretation of elevated FGF21 remains complex. In some contexts, FGF21 acts as an adaptive stress hormone, whereas in others, chronic elevation has been linked to BNIP3-associated mitophagy, glucocorticoid-mediated catabolic signaling, and reduced muscle mass (35–37). Importantly, at least in some experimental models, these atrophic effects do not appear to result solely from direct actions on skeletal muscle cells. Instead, they may be mediated in part through activation of the hypothalamic–pituitary–adrenal (HPA) axis and downstream glucocorticoid-responsive catabolic and anti-anabolic programs, including induction of Klf15 and FoxO1 and suppression of protein synthesis (37, 101). Therefore, in CKD-associated sarcopenia, FGF21 should not be presented as a uniformly harmful myokine. Rather, it is better interpreted as a context-dependent stress signal whose persistent elevation reflects metabolic burden and may contribute to muscle wasting under specific pathological conditions. Moreover, although FGF21 resistance is well recognized in obesity and diabetes, whether a comparable resistance state exists in CKD skeletal muscle remains unresolved.

#### 4.3.3 BDNF

BDNF also warrants conceptual refinement. Although evidence supporting its relevance to skeletal muscle is accumulating, it is best established biology remains centered on neural and neuromuscular regulation (41–45). Accordingly, BDNF is better framed not as a canonical anabolic myokine, but as a muscle-relevant mediator involved in metabolic adaptation, neuromuscular integrity, regeneration, and physical performance (41–45, 102, 103). Mechanistically, BDNF/TrkB signaling in skeletal muscle has been associated with AMPK-linked fatty acid oxidation and metabolic flexibility, as well as satellite cell differentiation, early regenerative responses, and maintenance of neuromuscular junction signaling (43–45, 102, 103). This profile is particularly important because CKD-associated muscle dysfunction is unlikely to be explained by proteolysis alone. Impaired neuromuscular quality, reduced regenerative capacity, and compromised metabolic adaptation are also likely to contribute substantially to weakness, frailty, and functional decline (42, 103).

In maintenance hemodialysis, lower circulating BDNF levels have been associated with poorer physical performance and a higher prevalence of severe sarcopenia and frailty-related phenotypes (42). The exercise responsiveness of BDNF further strengthens its translational relevance, as resistance training in hemodialysis patients has been reported to increase circulating BDNF together with improvements in handgrip strength, quality of life, and depressive symptoms (46). However, direct CKD-specific mechanistic studies remain comparatively limited. Thus, within the kidney-muscle axis, BDNF is best interpreted not as a simple anti-atrophic factor, but as a neuromuscular and metabolic support signal whose decline may reduce skeletal muscle resilience under uremic stress.

#### 4.3.4 GDF-15

In CKD-associated sarcopenia, GDF-15 is best interpreted as a stress-responsive mediator with potent biomarker potential and plausible though less direct muscle-catabolic relevance. Unlike myokines, GDF-15 is a divergent member of the TGF-*β* superfamily produced across a broad range of tissues, including the liver, lungs, and kidneys, in response to cellular stress and inflammation (24, 25). Circulating GDF-15 concentrations increase as kidney function declines and are associated with subsequent eGFR reduction and CKD progression, likely reflecting the combined effects of impaired renal clearance and systemic tissue stress (104). This clinical relevance is particularly evident in hemodialysis populations, where elevated GDF-15 levels are strongly associated with PEW and adverse clinical outcomes (26, 105).

Mechanistically, the best-established GDF-15 pathway is central rather than intramuscular. GDF-15 signals exclusively through the GFRAL-RET receptor complex, and GFRAL expression is overwhelmingly restricted to the caudal brainstem, particularly the area postrema and the nucleus tractus solitarius (27, 28, 106). This anatomical localization explains why GDF-15-related wasting is mainly mediated through appetite and energy-balance regulation rather than a defined muscle-autonomous receptor pathway. Experimental evidence indicates that GDF-15 suppresses appetite and may sustain or even increase energy expenditure in preclinical settings, which collectively contribute to a negative energy balance and systemic wasting (107).

However, current human data more strongly support GDF-15 as a robust indicator of cachexia risk and adverse outcomes rather than as a proven direct driver of skeletal muscle atrophy in CKD (26, 29–31). This distinction is critical because overstating causality would obscure the functional difference between an appetite-regulating mediator and a direct intramuscular signaling factor. Within this framework, GDF-15 should be regarded as an important component of the wasting phenotype and a promising biomarker, although its causal position in CKD-associated sarcopenia remains unclear.

### 4.4 Context-dependent pleiotropic signaling: IL-6

IL-6 illustrates how the biological meaning of a mediator depends on its source, timing, and physiological context. In sedentary patients with CKD, chronically elevated IL-6 mainly reflects systemic inflammation, including activated monocytes and other non-muscle sources, rather than the transient myokine response released by contracting skeletal muscle (108, 109). Although inflamed skeletal muscle may contribute to circulating IL-6 in some patients, it is unlikely to represent the predominant source (110). In this setting, sustained IL-6 exposure is associated with persistent JAK/STAT3 activation and induction of downstream inhibitory mediators such as SOCS proteins. These changes can impair insulin/IGF-1 signaling at the IRS-1/PI3K/Akt level and converge on catabolic pathways that promote muscle wasting (111, 112). By contrast, the acute and transient increase in IL-6 released from contracting skeletal muscle during exercise has a distinct physiological role. Rather than mirroring chronic inflammatory IL-6, this pulsatile myokine signal activates an AMPK-centered metabolic program that supports glucose handling and fatty acid oxidation (113, 114). It also contributes to anti-inflammatory counter-regulation by increasing IL-10 and IL-1 receptor antagonist (IL-1ra), while suppressing endotoxin-induced TNF-*α* production (115). In addition, exercise-induced IL-6 appears to support muscle remodeling, including satellite-cell-associated adaptation (116). Consistent with this adaptive function, IL-6 deficiency has been reported to impair muscle growth and satellite-cell proliferation in a STAT3-dependent manner (117). Thus, the apparent dual role of IL-6 reflects differences in source, timing, and exposure pattern rather than a true contradiction. Pathological IL-6 signaling in CKD is not equivalent to physiological exercise-induced IL-6 signaling. Therefore, the clinically relevant goal is not indiscriminate suppression of IL-6, but restoration of a healthier inflammatory and metabolic environment in which beneficial contraction-linked IL-6 responses can be preserved or re-established.

## 5 Integrated remodeling of the kidney-muscle axis in CKD

Taken together, the reviewed evidence supports an integrated model of the kidney-muscle axis in which CKD induces coordinated remodeling of endocrine, metabolic, inflammatory, and regenerative signaling networks. In CKD, reduced filtration capacity, uremic toxin retention, chronic inflammation, metabolic acidosis, endocrine disturbance, and physical inactivity together constitute major upstream drivers of this remodeling (9–16). These CKD-related stressors alter both the circulating abundance and biological effects of muscle-relevant mediators. Under this mechanistic paradigm, myostatin and activin A primarily reinforce proteolytic catabolism, whereas impaired IGF-1 and IL-15 signaling reflects failed anabolic compensation. GDF-15 and FGF21 link systemic stress to appetite dysregulation, energy imbalance, and wasting; reduced irisin and BDNF activity indicates diminished mitochondrial, regenerative, and neuromuscular resilience; and IL-6 illustrates the shift from adaptive exercise-associated signaling to chronic inflammatory signaling.

CKD-associated sarcopenia is unlikely to result from the dysregulation of any single myokine or circulating mediator. Current evidence therefore supports a systems-level interpretation of myokine dysregulation in CKD-associated sarcopenia, in which circulating mediators should be understood not merely as isolated biomarkers or causal factors, but as components of a clearance-distorted and inflammation-coupled signaling network.

## 6 Translational implications: biomarkers and therapeutic perspectives

These mechanistic insights hold profound translational implications, but they should be interpreted with caution. Evidence derived from CKD patient cohorts, CKD animal models, and non-CKD populations differs in relevance and should not be treated as interchangeable. Among currently available interventions, exercise remains the most clinically grounded strategy. Beyond improving muscle strength and physical performance, exercise may also modulate irisin, BDNF, and inflammatory signaling in ways that are biologically consistent with improved muscle resilience (46, 118, 119). This view is supported by meta-analytic evidence showing that impaired physical function, including low handgrip strength and slow gait speed, is associated with increased all-cause mortality in patients with CKD and end-stage kidney disease (120). Thus, exercise should not be regarded merely as supportive care, but as a physiologically coherent intervention capable of partially restoring the disrupted muscle endocrine and metabolic environment in CKD.

Biomarker development represents another important translational opportunity. Circulating irisin, GDF-15, FGF21, and multi-marker panels may help identify patients at increased risk of PEW, frailty, or progressive sarcopenia (26, 30, 38, 93). However, the interpretation of these biomarkers requires careful clinical and biological contextualization. Future studies should avoid relying on a single circulating mediator without accounting for CKD stage, dialysis modality, age, diabetes status, inflammatory burden, nutritional status, and physical activity. This is particularly important in advanced CKD, where plasma concentrations may reflect impaired renal clearance as much as altered production or secretion (24, 33, 47, 48).

Pharmacological targeting of myokine-related pathways requires even greater caution. In preclinical CKD models, inhibition of myostatin has produced encouraging improvements in muscle mass and function (52, 62). Similarly, ActRII-directed approaches, including bimagrumab, have shown favorable effects on body composition in non-CKD populations, although durable functional benefits and CKD-specific safety data remain insufficient (121). IL-6 receptor blockade and exercise mimetics are biologically interesting, but current evidence is insufficient to support broad clinical use for CKD-associated sarcopenia (93, 122). Overall, the available evidence does not suggest that a single “anti-myokine” therapy is imminent. A more realistic translational path is likely to involve biomarker-informed, multimodal interventions that combine exercise, nutritional optimization, inflammation control, and selective pathway targeting according to patient-specific biological profiles.

## 7 Current limitations and future directions

Several limitations in the current literature deserve attention. First, many human studies remain cross-sectional, limiting their ability to establish temporal relationships or causal pathways. Second, tissue expression, circulating concentration, and functional bioactivity are often interpreted interchangeably, although these dimensions may diverge substantially in CKD. Third, the tissue origins of several mediators remain incompletely defined, particularly in advanced CKD and dialysis-dependent states, where altered clearance and systemic inflammation further complicate interpretation. Fourth, sarcopenia is frequently examined without sufficient separation from overlapping but distinct conditions such as PEW, cachexia, frailty, and physical inactivity (7, 8, 10). These issues collectively limit the precision with which individual mediators can be assigned biomarker, adaptive, or causal roles.

Future studies should therefore prioritize longitudinal, stage-stratified clinical designs that can better define disease trajectories and mediator dynamics across the CKD spectrum. Compartment-aware approaches are also needed to distinguish tissue-level expression from circulating abundance and downstream biological activity. Multi-omic strategies integrating transcriptomic, proteomic, metabolomic, and functional phenotyping may help link molecular alterations to muscle mass, strength, endurance, and physical performance (12). Spatial transcriptomics and single-cell profiling may be particularly useful for identifying CKD-specific regulatory hubs and clarifying cell-type-specific signaling within skeletal muscle and related tissues.

Interventional studies should likewise move beyond lean mass as the sole or primary endpoint. Clinically meaningful outcomes, including muscle strength, gait speed, exercise tolerance, fatigue, quality of life, and patient-reported function, should be incorporated more consistently (120). This is especially important because CKD-associated sarcopenia reflects not only reduced muscle quantity, but also impaired muscle quality, neuromuscular integrity, metabolic flexibility, and regenerative capacity. Recent consensus recommendations emphasizing individualized, progressive, and combined aerobic-resistance exercise prescriptions across CKD stages provide a practical framework for future trial design (123). More broadly, future interventions should be designed to test whether modifying specific signaling pathways can produce durable improvements in function, rather than merely altering circulating mediator levels or body composition.

## 8 Conclusion

Myokines and muscle-relevant circulating mediators occupy a central position at the interface between kidney dysfunction and skeletal muscle wasting in CKD. The current evidence does not support reducing CKD-associated sarcopenia to a single pathogenic molecule. Instead, it points to a coordinated remodeling of the kidney-muscle signaling network characterized by heightened catabolic pressure, impaired anabolic responsiveness, mitochondrial dysfunction and bioenergetic remodeling, and diminished regenerative capacity. Future progress will depend on distinguishing biomarker value from causal function, clarifying tissue origin versus clearance effects, and linking molecular changes to meaningful patient-centered outcomes.
